# Progress of cancer research on astrocyte elevated gene-1/Metadherin (Review)

**DOI:** 10.3892/ol.2014.2231

**Published:** 2014-06-05

**Authors:** YONG HUANG, LE-PING LI

**Affiliations:** 1 Department of Gastrointestinal Surgery, Provincial Hospital Affiliated to Shandong University, Jinan, Shandong 250021, P.R. China; 2 Department of General Surgery, Zao Zhuang Municipal Hospital, Zaozhuang, Shandong 277101, P.R. China

**Keywords:** astrocyte elevated gene-1, metadherin, neoplasms, metastasis, chemoresistance

## Abstract

Tumor development is initiated by an accumulation of numerous genetic and epigenetic alterations that promote tumor initiation, invasion and metastasis. Astrocyte elevated gene-1 [AEG-1; also known as Metadherin (MTDH) and Lysine-rich CEACAM1 co-isolated (LYRIC)] has emerged in recent years as a potentially crucial mediator of tumor malignancy, and a key converging point of a complex network of oncogenic signaling pathways. AEG-1/MTDH has a multifunctional role in tumor development that has been found to be involved in the following signaling cascades: i) The Ha-Ras and PI3K/Akt pathways; ii) the nuclear factor-κB signaling pathway; iii) the ERK/mitogen-activated protein kinase and Wnt/β-catenin pathways; and iv) the Aurora-A kinase signaling pathway. Studies have established that AEG-1/MTDH is crucial in tumor progression, including transformation, the evasion of apoptosis, invasion, angiogenesis and metastasis. In addition, recent clinical studies have convincingly associated AEG-1/MTDH with tumor progression and poor prognosis in a number of cancer types, including hepatocellular, esophageal squamous cell, gallbladder and renal cell carcinomas, breast, non-small cell lung, prostate, gastric and colorectal cancers, and glioma, melanoma, neuroblastoma and osteosarcoma. AEG-1/MTDH may be used as a biomarker to identify subgroups of patients who require more intensive treatments and who are likely to benefit from AEG-1/MTDH-targeted therapies. The therapeutic targeting of AEG-1/MTDH may simultaneously block metastasis, suppress tumor growth and enhance the efficacy of chemotherapeutic treatments.

## 1. Introduction

Tumor development is initiated by an accumulation of genetic and epigenetic alterations, which promote tumor initiation, invasion and metastasis (1,2). During the development of human neoplasms, the hallmarks of cancer are acquired, including the sustainment of proliferative signaling, the evasion of growth suppressors, the resistance to cell death, the enabling of replicative immortality, the induction of angiogenesis, the activation of invasion and metastasis, genome instability and mutations, the tumor promotion of inflammation, the deregulation of cellular energetics and the avoidance of immune destruction. These hallmarks aid in our understanding of the diversity of neoplastic disease (3). With the continuous development of cancer research, our understanding of the molecular pathogenesis of cancer has been enhanced. Increasing efforts in cancer research have been focused on the study of oncogenes, tumor suppressors and signaling pathways. Since the development of a more in-depth understanding of the molecular etiology of carcinogenesis, specific oncogenes have been identified and have led to the generation of ‘molecular-targeted therapy’ (4). The development of a ‘pan-cancer’ therapy may be possible by targeting an oncogene which is ubiquitously overexpressed in almost all types of cancer and has a regulatory role in the multistep processes of carcinogenesis (5).

A novel gene that has been identified is AEG-1 [also known as Metadherin (MTDH) and Lysine-rich CEACAM1 co-isolated (LYRIC)], which has emerged as a potentially crucial mediator of malignant tumors, and a key converging point of a complex network of oncogenic signaling pathways (6,7). AEG-1/MTDH presents as an ideal target for the development of the next generation of effective cancer therapeutics.

## 2. Molecular cloning and structure of AEG-1/MTDH

AEG-1/MTDH was first reported by Su *et al* (8) in 2002 as a neuropathology-associated gene induced in human fetal astrocytes following human immunodeficiency virus-1 (HIV-1) infection or treatment with recombinant HIV-1 envelope glycoprotein (gp120). Subsequently, Kang *et al* (9) described the full-length cloning and functional characterization of AEG-1/MTDH. Next, Brown and Ruoslahti (10) used a phage expression library of complementary deoxyribonucleic acid (cDNA) from a mouse model of the lung metastasis of breast carcinoma to identify a lung homing peptide in AEG-1/MTDH that was overexpressed in metastatic breast cancer and promoted the homing of breast cancer cells to the lungs. In 2004, Britt *et al* (11) and Sutherland *et al* (12) separately reported a novel protein, LYRIC, that colocalized with the tight junction protein, ZO-1, in polarized prostate epithelial cells (11) and was present in the cytoplasm, endoplasmic reticulum (ER), perinuclear regions and nucleolus (12).

Full-length AEG-1/MTDH cDNA includes 3,611 bp, excluding the poly-A tail (9). The open reading frame from nucleotide 220 to 1,968 of AEG-1/MTDH encodes a single pass transmembrane protein (putative 582-amino acid) of ~64 kDa and with an isoelectric point of 9.33 (9). AEG-1/MTDH orthologues are reported in the majority of vertebrate species, but are not detected in invertebrates. With the exception of three putative lysine-rich nuclear localization signals (NLS), AEG-1/MTDH has no recognizable protein domains (13), and the presence of putative (monopartite or bipartite) NLS between amino acids 79–91, 432–451 and 561–580 suggests that it may enter into the nucleus (6).

The AEG-1/MTDH gene consists of 12 exons/11 introns, as identified through the use of a genomic BLAST search (http://blast.ncbi.nlm.nih.gov/Blast.cgi), and is located at 8q22 where cytogenetic analysis of human gliomas suggests recurrent amplification (9). In a number of malignancies, such as malignant glioma (14), hepatocellular carcinoma (HCC) (15) and breast cancer (16), the location is significant. In HCC and breast cancer, genomic amplification of AEG-1/MTDH has been found in patients (15,16). Several protein motif analysis methods have predicted that AEG-1/MTDH has a single transmembrane domain (9–12). With regard to whether AEG-1/MTDH is a type I b membrane protein (with a cytoplasmic C-terminal without a signal peptide) (9,11,12) or a type II protein (with an extracytoplasmic C-terminal) (10,11), considerable debate remains. In recent years, functional and clinical evidence significantly support an important function of AEG-1/MTDH in cancer development, including transformation, the evasion of apoptosis, invasion and metastasis (13). However, a generous amount of research is required to fully characterize the molecular and biochemical properties of AEG-1/MTDH.

## 3. Oncogenic functions of AEG-1/MTDH

**Figure 1:**
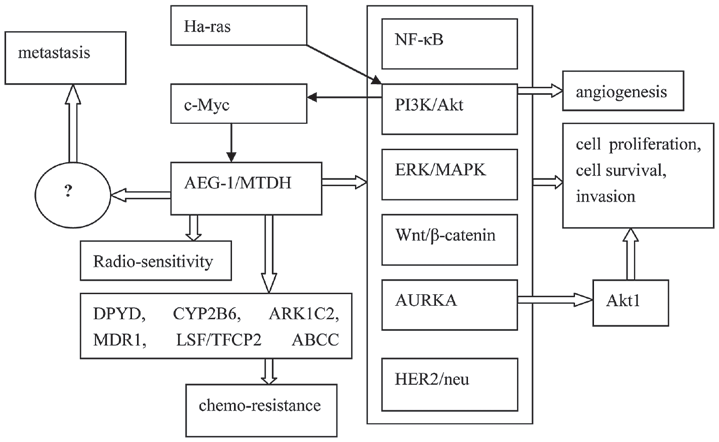
Hypothetical molecular mechanism of the action of AEG-1/MTDH. The thick white arrows indicate the regulation by AEG-1/MTDH, while the thin black arrows indicate the mechanisms that regulate AEG-1/MTDH. AEG-1, astrocyte elevated gene-1; NF-κB, nuclear factor-κB; MTDH, metadherin; MAPK, mitogen-activated protein kinase; AURKA, Aurora A kinase; DPYD, dihydropryimidine dehydrogenase; CYP2B6, cytochrome P450B6; ARK1C2, dyhydrodiol dehydrogenase; MDR1, multidrug-resistance gene 1.

AEG-1/MTDH mRNA is ubiquitously expressed at varying levels in all organs, as determined by multi-tissue northern blotting (9). The potential role of AEG-1/MTDH is to promote tumor progression and metastasis in human HCC cell lines and colorectal cancer (CRC) (17,18). AEG-1/MTDH localizes in the cell membrane, cytoplasm, ER and nucleus, and contributes to a group of signaling pathways, such as the PI3K-AKT, nuclear factor-κB (NF-κB), mitogen-activated protein kinase (MAPK) and Wnt pathways (19). AEG-1/MTDH is seminal in regulating proliferation, invasion, angiogenesis, metastasis and chemoresistance, as determined by ‘gain-of-function’ and ‘loss-of-function’ studies in human cancer cells and through the analysis of a transgenic mouse model (20). AEG-1/MTDH promotes tumorigenesis by modulating multiple signal transduction pathways and altering gene expression changes (Fig. 1).

### Integration of oncogenic pathways

Overexpression of AEG-1/MTDH synergizes with oncogenic Ha-Ras to enhance the soft agar colony formation of non-tumorigenic immortalized melanocytes and provides evidence of the tumor promoting activity of AEG-1/MTDH (9). AEG-1/MTDH expression is markedly induced by Ha-Ras, which activates the PI3K/Akt pathway leading to the binding of the transcription factor, c-Myc, to the E-box element in the promoter region of AEG-1/MTDH and the regulation of AEG-1/MTDH transcription (21). AEG-1 overexpression inhibits serum starvation-induced apoptosis by activating the Ras and PI3K-Akt signaling pathways (22). AEG-1/MTDH is also crucial in the carcinogenesis of non-small cell lung cancer (NSCLC) and inhibits apoptosis by enhancing the level of the antiapoptotic protein, Bcl-2, and activating the PI3K/Akt pathway (23). AEG-1/MTDH also downregulates the transcriptional activity of forkhead box (FOXO) 1 through the PI3K/Akt signaling pathway in MCF-7 and MDA-MB-435 breast cancer cells (24). In addition, AEG-1/MTDH is important in the aggressiveness of NSCLC through the activation of the PI3K-Akt and NF-κB signaling pathways (25).

Emdad *et al* (26) first reported that AEG-1/MTDH promotes the anchorage-independent growth and invasion of Hela cells by activating the NF-κB pathway. At the mRNA and protein levels, AEG-1/MTDH is upregulated during CRC development and aggressiveness (from normal mucosa to primary CRC, followed by lymph node metastasis and finally liver metastasis) through the NF-κB signaling pathway (27). AEG-1/MTDH contributes to the chemoresistance of cervical cancer cells by increasing autophagy and the activation of the ERK/NF-κB pathway (28). AEG-1/MTDH also modulates the BCCIPα protein levels in prostate tumor cells through an indirect mechanism involving the NF-κB signaling pathway (29). In malignant glioma cells, AEG-1/MTDH regulates invasion and migration through activation of the NF-κB signaling pathway (30), in which AEG-1/MTDH is involved in the lipopolysaccharide (LPS)-induced inflammatory response (31) and mediates the LPS-induced migration and invasion of breast cancer cells (32).

In human HCC cells, AEG-1/MTDH activates the MAPK pathways, including ERK and p38 MAPK, and is also associated with the Wnt/β-catenin pathway through the activation of the Raf/MEK/MAPK branch of the Ras signaling pathway (15). In proximal tubular epithelial cells, AEG-1/MTDH is important in TGF-β1-induced epithelial-mesenchymal transition (EMT) through activation of p38 MAPK (33). A recent study has suggested that AEG-1/MTDH contributes to the pathogenesis of diffuse large B-cell lymphoma mediated through regulation of the Wnt/β-catenin pathway (34).

Furthermore, in the carcinogenesis of acute myeloid leukemia (AML), a novel functional link has been revealed between AEG-1/MTDH and Aurora A kinase (AURKA) with regard to Akt1 activation (35). In human AML cells, AEG-1/MTDH overexpression is vital for the maintenance of the malignant state via upregulation of Akt1, which is mediated by AURKA activation (35). In breast cancer cells, AEG-1/MTDH facilitates cancer proliferation and invasion by upregulating HER2/neu expression (36).

### Angiogenesis and metastasis

AEG-1/MTDH overexpression converts non-tumorigenic human HCC cells into highly aggressive vascular tumors. In addition, AEG-1/MTDH modulates the expression of genes associated with invasion, angiogenesis, metastasis, chemoresistance and senescence, as determined by microarray analysis (15). AEG-1/MTDH has a dominant function in regulating oncogenic transformation and angiogenesis (37). AEG-1/MTDH expression is also increased in multiple cancers and is crucial in oncogenic transformation and angiogenesis (38–41). In a phage display study, Brown and Ruoslahti (10) identified that a lung homing domain (amino acids 378–440 in mice and 381–443 in humans) in AEG-1/MTDH was a mediator of 4T1 mouse mammary tumor cell adhesion to the lung vasculature, and suggested that AEG-1/MTDH is important in breast cancer metastasis. In CRC, Jiang *et al* (18) showed that AEG-1/MTDH is overexpressed in liver metastasis patients compared with patients without liver metastasis. In addition, AEG-1/MTDH may present as a potential novel biomarker for early liver metastasis. In a large proportion of epithelial ovarian cancer patients with peritoneal dissemination and/or lymph node metastasis, AEG-1/MTDH is overexpressed and is a novel predictor of metastasis (42). In summary, AEG-1/MTDH is crucial in lymph node metastasis (39,43–45) and contributes to tumor progression, including transformation, the evasion of apoptosis, invasion and metastasis (13).

### Chemoresistance

One of the important hallmarks of aggressive cancers is chemoresistance. Previous studies have suggested that AEG-1/MTDH contributes to a broad spectrum of resistance to various chemotherapeutics, including 5-fluorouracil, doxorubicin, paclitaxel, cisplatin and 4-hydroxycyclophosphamide (16,46–48). In human HCC cells, the gene expression profiles of overexpressed AEG-1/MTDH have been identified in several drug-metabolizing enzymes involved in chemoresistance, including dihydropyrimidine dehydrogenase, cytochrome P450B6, dihydrodiol dehydrogenase, ATP-binding cassette transporter 11/MRP8 and transcription factor LSF/TFCP2 (15). AEG-1/MTDH increases multidrug-resistance gene 1 (MDR1) protein expression, which facilitates the association between MDR1 mRNA and polysomes, leading to increased translation, the inhibition of ubiquitination and the resultant proteasome-mediated degradation of the MDR1 protein (47). The inhibition of AEG-1/MTDH may be an effective method in HCC chemotherapy (47). Bhutia *et al* (49) also showed that protective autophagy is the cause of AEG-1-mediated chemoresistance, and that the inhibition of AEG-1/MTDH results in a decrease in the protective autophagy and chemosensitization of cancer cells. Due to the multiple functions of AEG-1/MTDH in drug resistance, AEG-1/MTDH is a viable target as an anticancer agent for a wide range of cancer types (50).

Recent results have also indicated that AEG-1/MTDH affects the radiosensitivity of cervical cancer cells (51). In summary, it has become apparent that AEG-1/MTDH is an important oncogene, which is overexpressed in numerous human cancer types. Through a number of signaling cascades, AEG-1/MTDH is involved in several crucial aspects of tumor progression, including transformation, proliferation, the evasion of apoptosis, cell survival, migration and invasion, angiogenesis, metastasis and chemoresistance (52). Future studies are required to evaluate the correlation between AEG-1/MTDH function and signaling changes and interacting partners in order to highlight novel perspectives for AEG-1/MTDH as a significant target for the clinical treatment of various cancers.

## 4. Clinical-translational advances

In keeping with the role of AEG-1/MTDH in a number of different aspects of malignancy, AEG-1/MTDH has been found to correlate with tumor progression and poor prognosis in a number of cancer types, including HCC (17,53–58) and breast (59–64), prostate (65–67), glioma (68–70) and esophageal cancer (71) (Table I). These studies indicate that AEG-1/MTDH may be a powerful independent marker for poor prognosis and a viable target for anticancer therapeutics.

### HCC

Using transgenic mice with hepatocyte-specific AEG-1/MTDH expression, a previous study identified novel aspects of AEG-1/MTDH functions, including the induction of steatosis, the inhibition of senescence and the activation of the coagulation pathway to augment aggressive hepatocarcinogenesis (53). The results suggested that the expression of the AEG-1/MTDH protein was significantly higher in cancer cell lines with high metastatic potential, such as Sk-HEP-1 and MHCC-97H, than in those with low metastatic potential, such as HepG2 and Huh7. Additionally, AEG-1/MTDH has been shown to be closely associated with the abilities of the orientation chemotaxis and adhesion of HCC cells (17). In hepatitis B virus (HBV)-related HCC patients, AEG-1/MTDH expression has been found to significantly correlate with the American Joint Committee on Cancer (7th edition) (72) stage, T and N classification, vascular invasion and histological differentiation (54). In addition, patients with high AEG-1/MTDH levels have been found to exhibit poor survival rates compared with those with low AEG-1/MTDH levels (54). In HBV-related HCC patients, AEG-1/MTDH is a potential prognostic marker for overall survival (OS) and tumor progression, and is a chemotherapeutic target (54). In HCC tumors, the high expression of AEG-1/MTDH has also been found to correlate with microvascular invasion, pathological satellites, poor differentiation and tumor-node-metastasis (TNM) stages II to III. Furthermore, AEG-1/MTDH promotes HCC metastasis through induction of the EMT process (55). In a nude mouse model, the shRNA-mediated downregulation of AEG-1/MTDH resulted in reduced migratory capacity in HCC cell lines, and also reduced the number of abdominal and pulmonary metastases (55). AEG-1/MTDH overexpression and staphylococcal nuclease domain-containing 1 (SND1) lead to increased levels of RNA-induced silencing complex activity and contribute to hepatocarcinogenesis (56). Additionally, AEG-1/MTDH is a prognostic predictor of HCC following curative hepatectomy (57). AEG-1/MTDH overexpression has been identified in a high percentage of hepatitis B and C virus-positive HCC cases, and is key in the regulation of hepatocarcinogenesis (58). In summary, the AEG-1/MTDH gene is amplified in human HCC patients and promotes chemoresistance, angiogenesis and metastasis.

### Gastric cancer

The high expression of AEG-1/MTDH is observed in gastric cancer tissues. AEG-1/MTDH overexpression is associated with TNM Stage (TNM Classification of Malignant Tumors) (73), Ki-67 proliferation index and poor survival, and is an independent prognostic factor for gastric cancer in multivariate analysis (74). Inhibition of AEG-1/MTDH expression by specific small interfering RNA (siRNA) clearly inhibits SGC-7901 cell growth and enhances cell apoptosis by reducing the phosphorylation of AKT and glycogen synthase kinase-3β, and decreasing the levels of β-catenin, lymphoid enhancer binding factor 1 and cyclin D1 (74). Furthermore, the inhibition of cell proliferation and cell cycle arrest in gastric carcinoma SGC-7901 cells, mediated by the downregulation of AEG-1/MTDH expression, may be closely associated with changes in the expression of cell cycle-related proteins, including cdk2, cyclin D1 and p21 (75). AEG-1/MTDH overexpression is a useful prognostic factor in patients with gastric cancer, and the inhibition of AEG-1/MTDH may provide a novel therapeutic strategy for gastric cancer. However, in Iranian patients, AEG-1/MTDH mRNA expression was significantly elevated in 46.6% of examined tumor tissues, while its expression was low in others (36.6%) (76). There is only a marginal statistical difference between the AEG-1/MTDH gene expression in all tumor specimens compared with their paired non-tumor specimens, and no statistically significant association has been identified between the grades and types of tumors (76). At the transcriptional level, AEG-1/MTDH levels may be increased in gastric cancer tissue samples, but with considerable heterogeneity, and it may have the potential to be used as a target for diagnostic/therapeutic purposes only in a subset of patients (76). Therefore, the status of AEG-1/MTDH expression and its significance in gastric cancer remains unclear and requires further investigation.

### CRC

SND1 is a novel AEG-1/MTDH-interacting protein, and a functionally and clinically significant mediator of metastasis in breast cancer (77). In colon cancer tissues, a positive correlation has been identified between AEG-1/MTDH and SND1 expression by immunohistochemical staining; AEG-1/MTDH- and SND1-positive expression has been found to significantly correlate with nodal status, pathological stage and differentiation (78). Furthermore, OS time in colon cancer patients with positive AEG-1/MTDH and SND1 expression is significantly shorter than in those without AEG-1/MTDH and SND1 expression (78). The positive expression of AEG-1/MTDH and SND1 is an independent poor prognostic predictor in colon cancer, as observed by multivariate Cox regression analysis, and the increased expression of AEG-1/MTDH and/or SND1 is closely associated with the carcinogenesis, progression and prognosis of colon cancer (78). In predicting the prognosis of colon cancer, the coexpression of AEG-1/MTDH/SND1 may be a novel distinctive marker. During CRC development and aggressiveness, the AEG-1/MTDH mRNA and protein levels are upregulated and have been associated with tumor location and stage (27). Zhang *et* al (79) was the first to show that AEG-1/MTDH interacts with β-catenin in SW480 CRC cell lines and that AEG-1/MTDH expression closely correlates with the progression of CRC. The aforementioned studies also suggest that AEG-1/MTDH may be a potential therapeutic target in CRC.

### Esophageal squamous cell carcinoma (ESCC)

Immunohistochemical analysis of 168 ESCC specimens revealed that 47.6% of tumors exhibit high levels of AEG-1/MTDH expression (71). In ESCC patients, AEG-1/MTDH overexpression has been found to significantly correlate with the TNM stage, histological differentiation and a shorter survival time, and is an independent poor prognostic indicator, as determined by multivariate analysis (71).

### Gallbladder carcinoma (GBC)

High AEG-1/MTDH expression is present in highly invasive GBC-SD cell lines at the protein and mRNA levels, and in GBC samples (63.4%) compared with normal gallbladder mucosa. In addition, AEG-1/MTDH has been found to markedly correlate with differentiation degree, Nevin stage (80), Ki-67 expression and liver infiltration (81). In GBC patients, AEG-1/MTDH overexpression leads to a shorter survival time and is an independent prognostic marker, as determined by multivariate analysis (81). AEG-1/MTDH is a useful marker of GBC progression and may be a potential therapeutic target. The immunohistochemical analysis of 96 benign and 108 malignant lesions of the gallbladder revealed that the positive expression of erythropoietin-producing hepatoma-amplified sequence receptor A7 (EphA7) and AEG-1/MTDH is significantly higher in gallbladder adenocarcinoma than in benign lesions (82). In gallbladder adenocarcinoma, the positive expression of EphA7 and AEG-1/MTDH has been found to significantly correlate with differentiation, tumor masses, lymph node metastasis, invasion and OS, and is an independent poor prognostic predictor, as determined by multivariate analysis (82). The elevated expression of EphA7 and/or AEG-1/MTDH has also been found to closely correlate with the carcinogenesis, progression, clinical biological behavior and prognosis of gallbladder adenocarcinoma (82).

### Breast cancer

AEG-1/MTDH is expressed at low levels or is absent in the majority of normal human breast tissues, but is frequently overexpressed in ductal carcinoma *in situ* (83), breast cancer cell lines or breast tumors (9,10,16,59). The analyses of breast tumor samples collected in the USA and Japan revealed strikingly similar patterns of AEG-1/MTDH expression and clinical association (16,59). AEG-1/MTDH overexpression is significantly associated with estrogen receptor- and progesterone receptor-negative expression, a high nuclear grade, poor disease-free survival, a high Ki67 index, poor distant metastasis-free survival and poor OS (59). AEG-1/MTDH overexpression has a particularly negative impact on the prognosis of node-negative patients and is independently associated with poor disease-free and distant metastasis-free survival rates, as determined by multivariate analysis (59). Li *et al* (60) showed that AEG-1/MTDH may promote EMT in breast cancer cells in driving the progression of their aggressive behavior. Furthermore, Li *et al* (61) assessed the variants of the AEG-1/MTDH gene and their potential association with breast cancer susceptibility. The study discovered nine novel variants and found two variants to be associated with the susceptibility of breast cancer. AEG-1/MTDH has a dual function in promoting chemoresistance and metastasis, and is a key functional target of the 8q22 genomic gain that is frequently observed in breast cancer patients with a poor prognosis (62). In summary, AEG-1/MTDH overexpression contributes to an aggressive phenotype, leading to a poor prognosis in primary invasive breast cancer. Blocking AEG-1/MTDH and its regulated pathways is likely to be beneficial in breast cancer cells or tissues (63,64).

### NSCLC

In NSCLC cell lines and tissues, AEG-1/MTDH has a crucial function in the aggressiveness leading to a poor clinical prognosis (25) and promotes NSCLC metastasis by modulating matrix metalloproteinase-9 (MMP-9) expression (84). In NSCLC, AEG-1/MTDH overexpression has been found to significantly correlate with clinical staging, differentiation, lymph node metastasis and a shorter OS time (23). In L-78 cells, a previous study observed that AEG-1/MTDH siRNA treatment significantly upregulated caspase-3, markedly decreased Bcl-2, largely attenuated PI3K p110 protein expression and phosphorylated Akt (23). These results suggested that AEG-1/MTDH is crucial in the carcinogenesis of NSCLC and that it inhibits apoptosis by enhancing the level of antiapoptotic protein, Bcl-2, and activating the PI3K/Akt pathway (23).

### Renal cell carcinoma (RCC)

A markedly higher expression of AEG-1/MTDH was identified in eight cases of RCC tissue compared with the paired normal tissue from the same patient by quantitative reverse transcription polymerase chain reaction and western blot analysis (85). At the mRNA and protein levels, the expression of AEG-1/MTDH was also increased in four RCC cell lines, in contrast to normal tubular epithelial human kidney HK 2 cells (85). Furthermore, AEG-1/MTDH overexpression has been found to significantly correlate with tumor grade, clinical staging, T classification, metastasis classification and a shorter survival time, as determined by immunohistochemical analysis (85). A microarray study showed that high AEG-1/MTDH and p53 expression correlate with the prognostic parameters in RCC patients and may be associated with tumor progression (86). In conclusion, the AEG-1/MTDH protein is overexpressed in RCC and is important in tumor differentiation and progression.

### Prostate cancer (PC)

A significantly higher expression of AEG-1/MTDH has been identified in PC samples and cell lines compared with benign prostatic hyperplasia tissue samples and normal prostate epithelial cells (65,66). The knockdown of AEG-1/MTDH induces PC cell apoptosis through upregulation of FOXO3a activity, and also attenuates the constitutive activity of NF-κB and activator protein 1 (AP-1), with a corresponding depletion in the expression of NF-κB and AP-1-regulated genes. Knockdown also significantly decreases the cell invasion properties of PC-3 and DU145 PC cells (66). Recent findings have suggested that AEG-1/MTDH is overexpressed in PC cells and that cryptotanshinone exerts antitumor activity via inhibition of hypoxia inducible factor 1α, AEG-1/MTDH and vascular endothelial growth factor in hypoxic PC-3 cells (67).

### Glioma

Oncogenic AEG-1/MTDH is overexpressed in >90% of brain tumors and promotes gliomagenesis, particularly tumor growth and invasion, two primary characteristics of glioma (68). Lee *et al* (68) found that AEG-1/MTDH contributes to glioma-induced neurodegeneration, a hallmark of this fatal tumor, by regulating EAAT2 expression. Liu *et al* (69) suggested that AEG-1/MTDH expression significantly correlates with the clinicopathological stage of the glioma and contributes to glioma progression by enhancing MMP-9 transcription and promoting tumor cell invasiveness. AEG-1/MTDH expression is significantly elevated in >90% of diverse human brain tumor samples, including glioblastoma multiformes and astrocytic tumors, and in human glioma cell lines compared with normal brain tissues and normal astrocytes, as determined by western blot analysis and immunohistochemistry (70). AEG-1/MTDH may have a crucial function in the pathogenesis of glioma and may represent a viable potential target for malignant glioma therapy.

AEG-1/MTDH overexpression has also been documented in melanoma (9), neuroblastoma (48), osteosarcoma (87) and ovarian cancer (42,88,89), as well as in other tumors. However, certain controversy exists regarding the localization of the AEG-1/MTDH protein in the nucleus or cytoplasm of cancer cells, and the utility of nuclear or cytoplasmic AEG-1/MTDH to predict the course and prognosis of disease (20). Future studies are required to evaluate AEG-1/MDTH interaction partners in various cancer types and their significance in cancer progression. In summary, AEG-1/MTDH overexpression markedly correlates with advanced tumor characteristics and a poor clinical prognosis, and is a promising target for novel therapeutics.

## 5. Clinical translation and therapeutic targeting strategy

AEG-1/MTDH inhibition may be an effective anticancer strategy, considering that AEG-1/MTDH is overexpressed in a variety of cancer types and regulates the fundamental processes of carcinogenesis. AEG-1/MTDH overexpression can be utilized to identify subgroups of patients who require more intense treatments and who are likely to benefit from AEG-1/MTDH-targeted therapies. Patients with AEG-1/MTDH overexpression or amplification in different tumors may potentially suffer from metastatic recurrence and require close monitoring for clinical signs of relapse for early therapeutic intervention. High-risk patients require a higher dose of chemotherapy, the efficacy of which may be increased through the combination of chemotherapy with AEG-1/MTDH inhibition. Based on the molecular targeting of AEG-1/MTDH, novel cancer treatments consist of several possible avenues. AEG-1/MTDH-knockdown in MG-63 osteosarcoma cells significantly decreases ET-1 expression (at the mRNA and protein levels), cell invasion, MMP-2 expression and cell survival against cisplatin (87). Transfection of a recombinant plasmid containing pcDNA-AEG-1/MTDH-microRNA (miR)-4 significantly suppresses AEG-1/MTDH at the mRNA and protein levels by >69% in MDA-MB-231 breast cancer cells, and significantly inhibits proliferation, motility and migration compared with controls (90). Silencing AEG-1/MTDH expression by RNA interference was found to effectively reduce metastasis by 3- to 10-fold in a MDA-MB-231 xenograft model of breast cancer lung metastasis, and it was also shown to sensitize chemoresistant MDA-MB-231 breast tumors to paclitaxel or doxorubicin (16). Ward *et al* (91) observed that AEG-1/MTDH is a direct target of miR-375, and that AEG-1/MTDH-knockdown partially phenocopies the effects of miR-375 on the sensitivity to tamoxifen and the reversal of EMT in breast cancer. The treatment of tamoxifen-resistant breast cancer may be aided by the development of potential therapeutic approaches, such as the re-expression of miR-375 or the targeting of AEG-1/MTDH (91). In breast cancer cells, AEG-1/MTDH-miRNA may be important in the downregulation of proliferation, motility and migration, and must be used as a future potential small molecule inhibitor therapeutic targeting strategy. AEG-1/MTDH-knockdown decreases nuclear β-catenin accumulation and suppresses the migration and invasion of SW620 colorectal carcinoma cell lines (79). Adenoviral delivery of AEG-1/MTDH-targeting shRNA inhibits xenograft primary tumor growth in HCC mice (15). The inhibition of AEG-1/MTDH in neoadjuvant or adjuvant settings not only increases the response rate of chemotherapy, but also reduces tumor growth and the systemic spread of metastatic cancer (13). In MDA-MB231 human breast cancer cells, exposure to cadmium chloride affects AEG-1/MTDH expression (92). Current and future studies must focus on the translational aspects of AEG-1/MTDH, and on the understanding of AEG-1/MTDH function in physiological and pathological processes using transgenic and knockout mouse models in cancer. The knockdown of AEG-1/MTDH (32,33,41,45,51,93,94) or AEG-1/MTDH inhibition (92,95,96) can inhibit transformation, proliferation, the evasion of apoptosis, migration and invasion, metastasis, angiogenesis and chemoresistance. In summary, the functional characterization of AEG-1/MTDH as a novel protein with poorly-characterized functions is urgently required to realize its full therapeutic potential.

## 6. Conclusion and future perspectives

Accumulating evidence has demonstrated that AEG-1/MTDH has clear functions in the regulation of various physiological and pathological processes (97). AEG-1/MTDH is an extremely important molecule for the regulation of a variety of pathological and physiological processes by modulating the transcription and translation of factors involved in the signaling pathways (97). It is clear that AEG-1/MTDH has a decisive role in the process of tumorigenesis in multiple models, as determined by *in vitro* and *in vivo* studies and expression analysis (98). The multiple functions of AEG-1/MTDH highlight several important clinical implications. AEG-1/MTDH, as a ubiquitous biomarker for aggressive tumors, may be used for the routine screening of patients (13). AEG-1/MTDH is an important regulator in multiple aspects of cancer development and progression. Clinical and functional analyses suggest that the multifunctional gene, AEG-1/MTDH, is a potentially valuable target in cancer treatments. Chen *et al* (99) showed that the anti-AEG-1 autoantibody response may be a diagnostic biomarker for cancer patients with AEG-1-positive expression, and a possible inducer, with substantial immunity against AEG-1 by immunization boosting with AEG-1 vaccines. In a therapeutic model, the AEG-1/MTDH vaccine increased the chemosensitivity to doxorubicin and inhibited breast cancer lung metastasis (100). The AEG-1/MTDH vaccine in combination with chemotherapy may offer novel strategies for the treatment of cancer metastasis.

In conclusion, AEG-1/MTDH is a valuable diagnostic or prognostic biomarker based on its overexpression and correlations with disease staging and outcome throughout a wide range of cancer types. However, larger patient studies and a prospective investigation on the correlation between AEG-1/MTDH mRNA or protein in the blood and circulating tumor cells, and in urine and biopsy samples compared with clinical characteristics are urgently required.

## Figures and Tables

**Table I:** Studies on AEG-1/MTDH in a variety of cancer types.

Cancer types	First author/s, year (ref.)
HCC	Zhou *et al*, 2012 (17); Srivastava *et al*, 2012 (53); Gong *et al*, 2012 (54); Zhu *et al*, 2011 (55); Yoo *et al*, 2011 (56); Ahn *et al*, 2013 (57)
Gastric cancer	Jian-bo *et al*, 2011 (74); Zhang *et al*, 2013 (75); Baygi *et al*, 2012 (76)
CRC	Gnosa *et al*, 2012 (27); Wang *et al*, 2012 (78); Zhang *et al*, 2013(79)
ESCC	Yu *et al*, 2009 (71)
GBC	Sun *et al*, 2011 (81); Liu and Yang, 2013 (82)
Breast cancer	Kang *et al*, 2005 (9); Brown and Ruoslahti, 2004 (10); Hu *et al*, 2009 (16); Tokunaga *et al*, 2012 (59); Li *et al*, 2011 (60); Liu *et al*, 2011 (61); Wan *et al* (62) Kong *et al*, 2012 (63); Zhang *et al*, 2013 (64)
NSCLC	Ke *et al*, 2013 (23); Song *et al*, 2009 (25); Sun *et al*, 2012 (84)
RCC	Chen *et al*, 2010 (85); Erdem *et al*, 2013 (86)
PC	Thirkettle *et al*, 2009 (65); Kikuno *et al*, 2007 (66); Lee *et al*, 2012 (67)
Glioma	Lee *et al*, 2011 (68); Liu *et al*, 2010 (69); Emdad *et al*, 2010 (70)
Neuroblastoma	Liu *et al*, 2009 (48)
Osteosarcoma	Liu *et al*, 2013 (87)
Ovarian cancer	Li *et al*, 2011 (42); Li *et al*, 2012 (88); Yuan *et al*, 2012 (89)

AEG-1/MTDH, astrocyte elevated gene-1/Metadherin; HCC, hepatocellular carcinoma; CRC, colorectal cancer; ESCC, esophageal squamous cell carcinoma; GCB, gallbladder carcinoma; NSCLC, non-small cell lung cancer; RCC, renal cell carcinoma; PC, prostate cancer.
